# Influences and Barriers on Physical Activity in Pediatric Oncology Patients

**DOI:** 10.3389/fped.2016.00131

**Published:** 2016-12-19

**Authors:** Larrilyn Yelton, Shalini Forbis

**Affiliations:** ^1^Boonshoft School of Medicine, Wright State University, Dayton, OH, USA

**Keywords:** childhood cancer, pediatric oncology, exercise, influence, perspective, intervention, physical activity

## Abstract

**Objectives:**

To determine the influence of family, peers, school, and physicians on exercise in pediatric oncology patients and evaluate the barriers to physical activity (PA) levels in this population.

**Methods:**

A search of PubMed and Google Scholar resulted in 12 related articles. The articles were assessed for the influence of school systems, family, peers, self-efficacy, and physicians on exercise. Additionally, barriers and interventions to PA were also assessed. Limitations and research methodologies of each article were also evaluated.

**Results:**

Many school systems were unsure of expectations in regards to PA for their returning students with cancer. Most schools acknowledged willingness to increase exercise for these students; however, there is a communication gap between the medical field and the school system on what expectations should be. Family is associated with increased PA levels and healthier diets in this population with children preferring mothers as exercise partners more than fathers. While physician interventions have been shown to positively impact PA, it has been reported that physicians are not engaging in exercise counseling with their patients.

**Conclusion:**

Several issues and barriers related to PA in pediatric oncology population were identified. Studies have demonstrated that it is feasible to increase PA and self-efficacy in this population. Further research is needed to better understand and quantify these issues as well as further test the interventions that have been suggested in this review and have been successful in other pediatric populations.

## Introduction

In 2014, it was projected that the incidence of cancer would be 10,450 in children aged 0–14 and 5,330 new cases in adolescents aged 15–19 in the United States (1). There are an estimated 379,112 survivors of childhood and adolescent cancer as of 2010 in the United States (1). With improved cancer treatments, the 5-year relative survival rates have increased substantially over the past 40 years from approximately 49% in 1975–1979 to 68.1% in 2003–2009 (2). However, 62.3% of cancer survivors suffer from at least one chronic condition, and 27.5% have a severe or life-threatening condition by 29.2 years old (3). Some of these chronic conditions include obesity (4), type 2 diabetes mellitus (5), and cardiovascular disease (6). Physical inactivity is a risk factor for these chronic diseases. The American Cancer Society Institute recommends children engage in moderate to vigorous exercise such as running, aerobics, or heavy yard work for at least 60 min 5 days a week (7). Childhood cancer survivor (CCS) patients aged 9–18 that had a high rate of physical activity (PA) have been shown to have lower percent body fat mass, subcutaneous fat, abdominal visceral fat, and greater lean body mass compared with CCS patients that engaged in low PA (8). Götte et al. discovered that diagnosed pediatric patients undergoing cancer treatment in Germany self-reported less PA compared to before treatment began (9). This was especially apparent in patients staying in the hospital. Additionally, 60–70% of CCSs were found to be physically inactive when compared with their siblings or healthy controls (10). Thus, increasing PA in this population is critical.

Many cancer patients do not meet exercise requirements and suffer from comorbidities later in life that exercise helps to combat. Since this population is at risk for low exercise rates, it is imperative to understand the perspective and influence that family, friends, physicians, and educators have toward exercise and overcoming identified barriers. By understanding these influences, interventions can be incorporated to promote exercise in pediatric oncology patients.

Physical activity programs implemented in school systems have been reported to increase PA and overall fitness in children (11, 12). Since children spend a third of their day in school, it is important that the school system has knowledge of what to expect from these students. Additionally, school children with parental support engaged in more moderate–vigorous exercise when compared with their controls, highlighting the importance of having parental involvement in this process (13). Another influence on pediatric oncology patient PA is their physician. Physician advice and encouragement have been shown to bring about modifications in behavior and increased PA when compared to control groups (14).

The objective of this review is to evaluate the literature regarding current barriers to exercise identified by pediatric oncology patients. We also set out to ascertain, through thorough literature review, the various influences on this population including peer, parental, health-care, and the school administration and how these influences impact PA levels. Additionally, we will provide several ideas for how to overcome these barriers.

## Materials and Methods

For this review, PubMed and Google Scholar databases were searched using the following terms in various combinations: PA, exercise, motivation, influences, school, parent perspective, physician, pediatric cancer, and childhood cancer. Due to the limited literature on these subjects, inclusion criteria were broad. Only studies involving children under 18 years of age were included for review. Reference lists included in reviewed studies were used to obtain other relevant studies. The articles were assessed for the influence of school systems, family, peers, self-efficacy, and physicians on exercise. Barriers and interventions to PA were also examined. Additionally, the studies were evaluated for the methodologies used for data collection and limitations. Finally, additional intervention programs utilized in other pediatric populations were also included as suggestions that could be potentially beneficial in the pediatric population.

## Results

### Influences on Physical Exercise

#### Assessment Tools

A summary of all of the literature reviewed can be found in Table A1. Robertson and Johnson (15) mailed a questionnaire to all Local Education Authority regions (LEAs) in Scotland. Example questions include: “For a pupil who has returned to school during/after treatment for cancer how much would you expect that pupil to participate in PA (PE)?,” and “If extra PE were prescribed for a pupil returning to school during or after successful treatment for cancer, e.g. an additional 25 minutes of PE per day, could your school accommodate?” Several studies implemented the use of cross-sectional self-reported surveys (16–19). The Godwin Leisure Time Exercise Questionnaire was used by several studies to evaluate the PA levels in pediatric oncology patients (16, 17, 20). The Pediatric Quality of Life Inventory Version 4.0 was used to measure the health-related quality of life (HRQL) (16). PA metabolic equivalent (METs) hours per week were assessed by measuring oxygen consumption. To determine if respondents were receptive to diet and PA interventions, the questions “How interested are you in learning about weight control?” and “Would you want others to join in the intervention with you-if so, who?” were utilized (17). To assess the quality of the caregiver–recipient relationship, a Likert-type scale adapted from Lawrence Cross-sectional interviews was utilized in several studies (20–22). A Likert-like scale was used to assess family support for PA, peer support, perceived benefits to exercise, perceived barriers to exercise, and self-efficacy in regards to PA (20). The theory of planned behavior (TPB), which evaluates attitudes to gage the readiness for an individual to engage in a specific behavior, was utilized to further elicit motivation for exercise (19). Parenting practices, style, and behavior were analyzed through the implementation of an interview (22). The experiences and perspectives of parents were assessed through the use of an interview (21). The PA Stages of Change Questionnaire was implemented to gage the readiness of participants to alter PA behavior (18). This model groups individuals into five stages including the following: (1) precontemplation, where the individual does not engage in exercise and does not intend in changing within the next 6 months, (2) contemplation, where the individual does not exercise but is considering starting within the next 6 months, (3) preparation, where the individual currently engages in PA but not regularly, (4) action, where the individual exercise regularly but has begun so only within the last 6 months, and (5) maintenance, where the individual currently exercises regularly and has done so for more than 6 months. A previously used and validated self-reported survey from Abrahamson et al. was used to determine the beliefs, attitudes, and exercise counseling practices of pediatric oncologists in Alberta.

#### School Systems’ Perspective and Influence on Exercise

Only one study has been reported, which evaluated academic expectations, past experiences, and physical expectations of pediatric oncology students returning to school in Scotland. Over half (*n* = 224) of LEAs responded to the survey. Additionally, only 20% of schools that responded to the survey reported they had experience with one or more cancer patient. LEAs did not anticipate increased PE for cancer patients returning to school. The majority of LEAs were uncertain of what to expect in regards to the PA of the child and believed there would be medical guidance provided for each child upon school reentry. The sources of information reported by the schools about education on the child included parents, hospital nurses, and students in descending order of commonality. Almost all schools were willing to increase PA if prescribed for these patients, preferably substituting PA for other lessons or at the start or end of the day. The largest barrier to overcome to accommodate increased PE was supervision difficulties. Other challenges reported after these children returned to school included: behavior issues, extra emotional support needed for the child and staff interacting with the child, and other student’s being “overly protective” upon the return of their peer. Primary schools were unsure how exercise would impact academic progress; in contrast, secondary schools thought increased PE would increase academic performance. Both primary and secondary schools believed increased PE would enhance social development of the returning pupil.

While this study had a substantially large sample size and decreased bias by sampling the entire population in Scotland, the questionnaire was sent *via* mail. Problems that may have arisen from this method of distribution include: the lack of ability for participants to ask questions if parts of the survey were ambiguous/not understood and that the survey was not piloted prior to the start of the study. Additionally, this article is 13 years old, which may limit the generalizability to current populations due to the vast increase in technology that now makes access to education on any topic readily available. Moreover, a further limitation to this study is the location and the school systems studied being in Scotland, which may not be generalizable to the United States School systems. Also, this study did not identify the role of the individual completing the survey; therefore, different administrative roles may have more or less knowledge on exercise guidelines in pediatric oncology patients.

In summary, this one study demonstrated a self-reported willingness on the part of schools to accommodate pediatric oncology patients returning to school; however, as reported by the majority of schools, there is a vast educational gap on what should be expected and implemented for these children in regards to PA and how PA will impact students’ academic performance. Previously used school-based educational interventions may help to overcome many of these gaps/barriers. For example, it was shown that implementing an after-school exercise program in third grade students without cancer was not only feasible but also resulted in increased physical fitness and body composition (23). Additionally, schools implementing PA time during school have been reported to increase PA, especially when parents were involved (13). Both primary and secondary schools reported increased PE modifications are feasible and can be implemented. To encourage the implementation of these interventions, more communication is needed between the medical team and school system. This may be done through the implementation of reentry school programs that have been previously reported to be successful (24). While schools were concerned how PA would impact academic performance, several studies have revealed that exercise does not impair academic performance (25) but rather promotes performance, concentration, memory, and classroom behavior (26). Further research should investigate intervention programs in schools that not only increase PA but also educate school administration on physical and academic expectations in these students.

#### Family Influence

Family has been reported to be an influence on PA behavior for a number of years (27). Norris et al. (16) reported survivor total PA and mother total PA metabolic equivalents (PA METs) were significantly correlated as were father–sibling total PA METs; however, sibling–survivor PA did not have a significant correlation. While exercise rates were not different between siblings, cancer patients exercised less intensely when compared to siblings, findings that were similar to other reports (10). Interestingly, parents typically rated their cancer-surviving child lower for HRQL than the child rated himself or herself in areas of school functioning and emotional functioning. Siblings rated their HRQL lower than their parents’ proxy rating. This indicates a gap between perspectives of children and parents. This gap may explain why parents of cancer survivors often are less demanding when it comes to scholastics and other situations to avoid upsetting their child with cancer (21). Substantiating the contrasting HRQL rating between parents and children, parents HRQL corresponded with PA levels: CCSs were rated as having a lower HRQL and had lower PA (16).

Another way parents can influence their children is by engaging in physical exercise with them, behavior modeling, and assuming more authoritative parenting styles (22). A study by Santa Maria and colleagues reported that children displayed better exercise habits if their parents exhibited greater authoritative styles. Additionally, parents who exercised with their children, thus implementing good parent modeling, had children that were more likely to engage in exercise. Patients reported it was more difficult to exercise and maintain a healthy diet and weight when their parents did not model these behaviors and felt it was hypocritical (22). Some mothers reported being protective and did not want their child to obsess over losing weight due to everything else they had recently been subjected to. Finally, children reported it was difficult to exercise with the lack of a supportive home environment. Limitations of this study include the fact that it was completed only in central nervous system (CNS) tumor survivors, which may not be representative of families of children with other cancer types.

It has been reported for a number of years that exercise partners can increase the engagement in PA (28). Additionally, parental exercise engagement has been associated with child exercise engagement (29). Badr et al. (17) evaluated this correlation by asking parents and children “would you want others to join in the intervention, if so, who?” (17). They found that mothers were both more likely to exercise and were reported by their child with cancer to be more preferred (45.9%) as exercise partners when compared to fathers (30.2%). Some limitations of this study include the fact that 80% of participants were mothers and 72% of respondents were white. Therefore, this may not be a complete representation of the populations. Additionally, participants were financially compensated for their time, which may have also affected the population of respondents. Gilliam et al. also found that family support had both a direct and indirect effect on moderate and vigorous PA engagement (20). They reported that the younger the child was at diagnosis, the more family support they received. Given the support of this influence, interventions, such as those developed in Cousins et al., can be implemented to increase PA in this population. In this study, families attended a class and obtained educational material about exercise and diet. Participants that attended the class as a family had the highest reduction in weight-loss compared to those who just received information and did not have the class. The importance of cultural engagement was also reported in this study (30).

#### Social and Peer Support

In addition to school being an important means to an education and PA, social and peer support can also influence PA in children. As mentioned in Badr et al. exercise partners and socialization can impact PA levels (17). In Gilliam et al. peer support was analyzed and found to have a positive association with PA and self-efficacy (20). Friends were also identified as a critical part of support in Keats et al. (19). A limitation of this study was the lack of assessment on how much the childhood cancer patients’ peers were exercising in total. Furthermore, this study was only performed in one hospital and thus is not representative of the entire population.

A recent protocol was published in order to educate schools and peers about a child with cancer’s condition and expectations with PA (31). It requires peers of the child with cancer to act as ambassadors for the child to improve PA. This study would not only encourage social interaction but also would enhance the school’s understanding of what to expect and allow administration to develop proper PA interventions for these students.

#### Physician Influence

Many pediatric oncology patients have reported that doctors do not engage in educating pediatric oncology patients and families about exercise (19, 32). Unfortunately, this is a recurring theme nationwide, even in patients who are healthy or who have other chronic illnesses (33, 34). Keats et al. in a study of 21 pediatric oncology physicians in Alberta, Canada, found that of physicians that responded (*n* = 11) all believed exercise to be important for their patients (35). Fifty percent believed there was no adverse risk with PA. Most physicians reported they counseled their patients on exercise but did not believe their patients followed the recommendations. Physicians reported very few patients inquired about exercise recommendations. The major barriers to PA counseling for physicians were lack of knowledge/resources and lack of time. The time spent on PA counseling was 1–5 min by 73% of responding physicians. All physicians said they verbally counsel patients, while few offered written materials or would refer to a specialist. Only one physician provided details for his or her recommendation of the frequency, intensity, time, and type of PA. A limitation of this study is the small sample size and the lack of generalizability to the United States. However, this study either suggests a knowledge gap in recommendations for pediatric oncology patients or suggests physicians prescribe different recommendations to various patients, which should be elucidated in further studies. The only current guidelines available are those by the Children’s Oncology Group for pediatric cancer survivors for healthy living and the American Cancer Society’s Guidelines to for PA to prevent cancer (36). Future studies should address the knowledge physicians have of these current recommendations and guidelines should be developed as well as resources for patients and their families.

Götte et al. reported that pediatric oncology patients and their families value their doctors’ opinions (32). Several physician intervention programs in healthy children have been shown to be successful in increasing PA in patients (14, 37). By adding just 3–5 min of exercise counseling with the physician and having a health educator call for a few minute “booster” 2 weeks later, exercise was shown to improve by 37 min per week in patients without cancer that received this counseling intervention (14). This is an intervention that pediatric oncologists, with the help of their medical team, could implement in order to increase the PA of their patients. Few studies have evaluated the pediatric hematologist/oncologist perspective on exercise in this population. No studies have assessed physician knowledge of the current PA guidelines as stated by the American Cancer Society. Additionally, few studies have been performed evaluating the pediatric hematologists’/oncologists’ communication about PA with patients and families. Thus, more research should be completed in this area so physicians can better counsel their patients on PA.

#### Self-Efficacy and Attitudes Influence

Self-efficacy – the confidence an individual has in his or her abilities to successfully complete a task – has also been reported to be positively associated with exercise in many different age-groups (38). In the study by Gilliam et al. higher self-efficacy increased the likelihood of engaging in moderate to vigorous PA (20). One intervention that could improve self-efficacy and PA is the Lifestyle and Education for Activity Program (LEAP). Dishman et al. (39) reported that self-efficacy was promoted with the LEAP program that was implemented in adolescent girls without cancer. This increased self-efficacy, in addition to the program itself, increased PA (39). The LEAP program was a 2-year intervention that increased PA in schools and was supported by the entire school system. Implementations like this would not only benefit children with cancer but also all children attending school. Another intervention that could be implemented in pediatric oncology patients is exercise goal-setting, which mediates the relationship between self-efficacy and PA (40).

In addition to self-efficacy, attitudes also impact engagement in PA (41). The TPB is one way in which attitudes or intentions are analyzed. It states that “attitudes toward the behavior, subjective norm, and perceived behavior control can gage the readiness of an individual to engage in that behavior.” In this study, Keats et al. assessed various beliefs and attitudes to find factors that correlated significantly with PA. Significant correlations included: self-efficacy, affective attitude (boring–interesting, enjoyable–unenjoyable), and behavioral beliefs (i.e., staying fit and looking good). There was no correlation between normative beliefs – the impact of the beliefs of those around the participant have on the participant’s behavior – and PA. This study was completed in Canada, which may not be generalizable to the United States. Additionally, patients were asked about their parent’s attitude on PA to determine how that impacted the child’s attitude toward PA; however, it was not assessed whether or not patients ever actually had conversations about these topics with their parents. Another limitation is the data from all of the questions were not reported. Some items in normative beliefs may have skewed the data to being less significant for various individuals (i.e., a parent’s belief may have more influence than a coach on a child). When PA readiness was assessed in pediatric oncology patients, it was found that the majority of participants were in the preparation stage and many others were in either the precontemplation or contemplation stage (18). In order to increase the engagement in PA, self-efficacy, attitudes, and readiness for PA need to be maximized in this population.

### Barriers to PA and Interventions to Resolve Them

#### Assessment Tools

Arroyave et al. implemented the use of a cross-sectional questionnaire with the implementation of the Likert-scale and open-ended questions to elicit barriers to exercise. Götte et al. implemented interviews to assess barriers to exercise qualitatively. Self-confidence was measured using PA self-efficacy, which asks the participants’ level of sureness on a variety of tasks (42). Quality of life was assessed using the Pediatric Quality of Life Inventory.

#### Barriers

Barriers to exercise are another reason this population often does not engage in recommended levels of PA. The interviews conducted by Götte et al. in pediatric cancer survivors reported that the average patient only engaged in 24.3 min of exercise with the majority occurring in the cancer ward. Barriers included physical aspects such as: fatigue (15, 18, 43), concern for increased risk of infection (18, 32), side effects of treatment, GI problems, pain, dizziness, and weakness (32). Many patients associated these barriers with chemotherapy. Some of these barriers, such as fatigue, may be improved by exercise (44). Psychological barriers included: lack of energy, bad moods/not feeling like it, preference for other things like staying in bed (32, 43), concern for injury, fear of soreness and sweating (43), falling behind academically (15, 18), and preference for sleeping to not feel side effects (32). Organizational barriers included: lack of time, lack of sports equipment, bad weather, lack of ideas (32, 43), lack of space for physical activities (especially when on infusion), and no one to play with (32). A limitation of this study was the small sample size.

One suggestion by patients to resolve some of these issues was to have access to an all-day sports therapist (32). Additionally, boredom and lack of ideas for PA can be resolved with the implementation of interventions such as those used in the LEAP program, which has been reported to increase enjoyment of physical exercise (45). A recent study showed that supervised-, targeted-exercise interventions increased PA more than encouraging participants to continue with exercise regimes at home suggesting that physical therapy-targeted interventions may be more efficacious than home-based programs (31). Additionally, an adventure-based training program and health education program has also been shown to increase PA and self-efficacy in this population, which may prevent several barriers identified such as boredom and lack of sports equipment. Future studies should address how different interventions can overcome these identified barriers.

## Conclusion

In conclusion, this review identified a number of issues related to appropriate PA for pediatric oncology patients. Barriers to exercise identified were psychological, organizational, and physical. Additionally, schools and educators do not demonstrate experience with CCSs and do not know what to expect from patients in regards to exercise. Family support is associated with both increased self-efficacy and increased activity levels with child preference for mothers as exercise partners. In other populations, family has been shown to be the most important social factor affecting weight-loss and exercise. While patients and families report to value their physicians’ opinion in regards to PA, it has been reported that physicians are not providing exercise counseling to their patients and their patients’ families. There have been no new updated studies on the influences of pediatric cancer or exercise counseling by physicians; thus, future research is needed to ascertain if pediatric oncologists are participating in exercise counseling and how interventions can be developed to overcome barriers.

## Author Contributions

LY completed and synthesized the literature search and drafted the manuscript. SF contributed to the manuscript and reviewed, critiqued, and revised the manuscript.

## Conflict of Interest Statement

This manuscript was not funded by any organization. None of the authors have any financial interests invested in this project. The authors declare no conflicts of interest.

## Appendix

**Table A1:** **Table includes the design type, demographic data, goals of each article, and main findings of each article**.

Reference	Design	Demographics	Goal	Results
Arroyave et al. (43)	Cross-sectional survey	*N* = 33 childhood survivors Children <18 years old, Muenster, Germany	Identify barriers to exercise in this population	Common barriers included fatigue, too busy, bad weather, do not belong to gym, preference for TV, do not like to sweat

Badr et al. (17)	Cross-sectional mail survey	*N* = 106 survivors only *N* = 50 parent only *N* = 114 parent and survivor M.D. Anderson <18 years old	Assess the relationship between parent and patient BMI, PA, quality of the patient–parent relationship, and desire to exercise together	No relationship between survivors and parents for BMI, patients had lower BMI than parents, results suggested parents and siblings do not exercise together currently but both have a desire to, only 1/3 of survivors meet national guidelines

Chung et al. (18)	Cross-sectional survey	*N* = 128 Pediatric oncology patients 9–16 years old, Hong Kong *N* = 128 survivors	Assess the current activity levels in patients, identify the readiness for PA change, assess barriers to exercise	Patients reported participated in less exercise post diagnosis, fear of academic faltering was the largest barrier reported, the majority of patients were in the preparation stage of PA readiness

Van Dongen-Melman et al. (21)	Cross-sectional interview	*N* = 87 parents of children 8–13 years old	To evaluate the experiences and perspectives of parents with childhood cancer aged 8–13 years old	Parents attempted to prevent unhappy or stressful situations by setting less demands. Parents were protective and preventative of children participating in research due to the potential of distress. Parents also succumbed to child’s wishes in order to prevent stressful situations

Gilliam et al. (20)	Cross-sectional phone survey	*N* = 105 survivors 8–16 years old children of Alabama	To identify the level of PA in childhood cancer survivors and the influence of self-efficacy, family and peer support, perceived benefits, and barriers on PA levels in cancer survivors	29% of survivors did not meet ACS recommendations for PA. Higher family and peer support, more perceived benefits, less perceived barriers, and higher self-efficacy increased the likelihood of exercise

Götte et al. (9, 32)	Cross-sectional interview	*N* = 40 survivors Pediatric patients ≤4 years old, Germany	Identify the values and beliefs toward exercise during treatments, exercise barriers and motivation, encouragement of parents and physicians	All patients thought exercise would be beneficial. Barriers included physical (side effects of treatment, dizziness, fatigue), psychosocial (bad moods, lack of energy), and organizational (lack of time, space, and ideas). Parents were supportive, not supportive, or apathetic. Physicians did not give any opinion on exercise

Keats et al. (19)	Cross-sectional survey	*N* = 36 – elicitation questionnaire *N* = 9 – determinants questionnaire CCS 15–20 that were diagnosed between 11 and 19 years old	To examine the utility of the theory of planned behavior (TPB) in order to further under motivation for exercise in the adolescent cancer survivor population	The factors that correlated significantly with PA included self-efficacy, affective attitude (boring–interesting, enjoyable–unenjoyable), and behavioral, beliefs. Normative beliefs had no significant correlation with PA. Self-efficacy and intention were useful for predicting PA. Affective and instrumental attitudes were predictors of intention to be physically active. Physicians were not deemed as a source of support

Keats et al. (35)	Cross-sectional survey	*N* = 21	Examine attitudes, counseling practices, and beliefs of pediatric oncologists in Alberta, Canada	PA in pediatric oncology patients was important to all responding doctors. Half of doctors did not believe PA had adverse risk in the pediatric population. Few physicians could write down the prescription for PA for their patients, few believed patients followed their recommendations for PA

Li et al. (42)	Cross-sectional randomized control intervention	*N* = 71 pediatric oncology patients 9–16 years old *N* = 37 control Intervention-34 Hong Kong	Identify the impact of adventure-based training and health education on exercise levels, self-efficacy, and quality of life	Experimental group had higher levels of self-efficacy and PA, but no statistically significant difference was found in quality of life when compared to the control group

Santa Maria et al. (22)	Cross-sectional survey	*N* = 8 survivors and mothers CNS tumor survivors, Texas	Assess the impact parenting style, behavior, and practices has on PA in childhood cancer survivors	Homes with parent modeling of exercise and healthy eating were more likely to follow parental examples, authoritative parenting style had more healthier exercise patterns

Norris et al. (16)	Cross-sectional survey	*N* = 17 pediatric cancer survivors *N* = 10 siblings *N* = 33 parents CCS 10–17 years old, Alberta, Canada	Assess the physical exercise and health-related quality of life in pediatric patients and their families and determine any correlations	Mothers were more likely to engage in PA than fathers. Survivor–mother total PA and METs correlated, sibling–father METs total PA METs correlated, and there was no correlation between siblings and survivors. Parents assumed survivor children had lower HRQL and emotional functioning than siblings. Siblings rated their HRQL lower than parents, while survivors rated social functioning higher than their parents did. Physical, psychosocial health, social health, and school functioning were in agreement with survivor–mother

Robertson and Johnson (15)	Cross-sectional survey	*N* = 308 primary schools *N* = 93 secondary school Primary and secondary school in Scotland	Identify knowledge/willingness of school to assist with cancer patients in exercise	Majority of schools did not know what to expect of cancer patients in regards to exercise, some thought exercise would be beneficial, half believed more exercise could be incorporated
